# A checklist of macroparasites of *Liza haematocheila *(Temminck & Schlegel) (Teleostei: Mugilidae)

**DOI:** 10.1186/1756-3305-1-48

**Published:** 2008-12-31

**Authors:** Aneta Kostadinova

**Affiliations:** 1 Institute of Parasitology, Biology Centre of the Czech Academy of Sciences, Branišovská 31, 370 05 České Budějovice, Czech Republic; 2 Central Laboratory of General Ecology, Bulgarian Academy of Sciences, 2 Gagarin Street, 1113 Sofia, Bulgaria

## Abstract

**Background:**

The mugilid fish *Liza haematocheila *(syn. *Mugil soiuy*), native to the Western North Pacific, provides opportunities to examine the changes of its parasite fauna after its translocation to the Sea of Azov and subsequent establishment in the Black Sea. However, the information on macroparasites of this host in both ranges of its current distribution comes from isolated studies published in difficult-to-access literature sources.

**Materials and methods:**

Data from 53 publications, predominantly in Chinese, Russian and Ukrainian, were compiled from an extensive search of the literature and the Host-Parasite Database maintained up to 2005 at the Natural History Museum, London.

**Results:**

The complete checklist of the metazoan parasites of *L. haematocheila *throughout its distributional range comprises summarised information for 69 nominal species of helminth and ectoparasitic crustacean parasites, from 45 genera and 27 families (370 host-parasite records in total) and includes the name of the parasite species, the area/locality of the host capture, and the author and date of the published record. The taxonomy is updated and the validity of the records and synonymies are critically evaluated. A comparison of the parasite faunas based on the records in the native and introduced/invasive range of *L. haematocheila *suggests that a large number of parasite species was 'lost' in the new distributional range whereas an even greater number was 'gained'.

**Conclusion:**

Although the present checklist provides information that will facilitate future studies, the interesting question of macroparasite faunal diversity in *L. haematocheila *in its natural and introduced/invasive ranges cannot be dealt with the current data because of unreliability associated with the large number of non-documented and questionable records. This stresses the importance of data quality analysis in using host-parasite database and checklist data.

## Background

*Liza haematocheila *(Temminck & Schlegel, 1845) (syn. *Mugil soiuy *Basilewsky, 1855 see *e.g. *[1-3]), native to the Western North Pacific, provides an intriguing case for studying the effect of host translocation on parasite fauna because of the disparity of its introduced and natural range and the presence of a number sympatric mullet species in the former. *L. haematocheila *established a successful breeding population in the Sea of Azov in the early 1980s after numerous deliberate introduction attempts to support commercial fisheries [4] and is now the most abundant mugilid species present [5]. This species has already been established in the north-eastern Black Sea where it is a subject to commercial fisheries in Russia and the Ukraine since 1995. A small stock has been exploited along the Turkish coasts since the 1990s. The environmental conditions in the new species range appear to favour this species whose growth rate exceeds that of the native mullet species [6]; its expansion in the Black Sea coincides with a decline in the native mullet species which it apparently replaces [5]. Furthermore, *L. haematocheila *has been recorded in the Aegean Sea and Starushenko & Kazanski [7] predicted its ongoing invasion towards the Western Mediterranean.

The idea that the translocation and introduction of hosts into areas outside their natural distributional range results in a reduction in the number of their parasite species or a loss of their original parasite fauna was first formulated in the classical works of Dogiel [8,9]. This generalisation was reinforced by Kennedy & Bush [10] and has recently gained empirical support in tests of the 'enemy release' hypothesis at the host population level [11,12]. However, studies on teleost host-parasite systems are notably few in the marine environment (see Torchin *et al. *[11] for a review). An important prerequisite for such studies is the delineation of the parasite faunas in both fish native and invasive range; this incorporates the use of an updated taxonomy and quality assessment of the existing data.

This paper presents the first checklist of helminth and crustacean parasite species recorded in *L. haematocheila *in which largely scattered host-parasite records written in Chinese, Russian and Ukrainian and from difficult-to-access literature sources are compiled in an attempt to provide a biogeographical framework for future research on the role of parasites in the possible outcomes of invasion of *L. haematocheila *in the Mediterranean. The taxonomy is updated and the validity of the records and synonymies are critically evaluated and discussed.

## Methods

Data for the checklist were compiled from an extensive search of the literature and the Host-Parasite Database maintained at the Natural History Museum, London [13]. The main limitations of the data are related to the very small number of documented records (*i.e. *providing supportive evidence for the species identification *e.g. *description and/or figure/metrical data); these are indicated in the checklist (Additional file 1). Another feature of the data is that many records represent re-iterations of previous records (but without citations in a large number of cases especially in the Sea of Azov and Black Sea). The bias due to re-iteration of own records in a number of abstracts/species lists is obvious in the latter region where three teams have published on the parasite fauna of *L. haematocheila*, *i.e. *Dmitrieva and Gaevskaya [14-18], Maltsev and colleagues [19-26] and Sarabeev and Domnich [27-34]. The records of these teams alone come from 21 publications (out of 27 for the Sea of Azov and the Black Sea).

## Results

Altogether 69 species of helminth and ectoparasitic crustacean parasites from 45 genera and 27 families have so far been reported in *Liza haematocheila*; of these, 8 are identified to generic level (Additional file 1). Digeneans represent the most diverse group of parasites in this host (36 species belonging to 25 genera and 10 families), whereas the other higher-level taxonomic groups are poorly represented (11 monogeneans, 9 nematodes, 6 copepods, 4 acanthocephalans, 2 cestodes, and 1 isopod).

The parasite fauna of *L. haematocheila *appears to be more intensively studied in the Sea of Azov and the Black Sea (a total of 281 host-parasite records) as compared to Western North Pacific (89 records). At first glance this more than three fold difference indicates that differential study effort might have biased the data on faunal richness in the two areas. However, a reverse difference of the same magnitude is observed when only documented records are considered (*i.e*. 12 *vs *37 records, respectively, see Additional file 1). A total of 33 species (documented records for 24) were found to parasitize *L. haematocheila *in its natural (Western North Pacific) range and 44 species (documented records for 8) were recorded in its introduced/invasive range (Sea of Azov/Black Sea). Of these nine were reported in both areas: the monogeneans *Ligophorus chabaudi *Euzet & Suriano, 1977, *Ligophorus kaohsianghsieni *(Gusev, 1962), *Ligophorus llewellyni *Dmitrieva, Gerasev & Pron'kina, 2007, *Ligophorus pilengas *Sarabeev & Balbuena, 2004, *Gyrodactylus mugili *Zhukov, 1970, *Gyrodactylus zhukovi *Ling, 1962, *Microcotyle mugilis *Vogt, 1878 and the acanthocephalans *Neoechinorhynchus agilis *(Rudolphi, 1819) and *Acanthogyrus *(*Acanthosentis*) *tylosuri *(Yamaguti, 1939).

**Figure 1:**
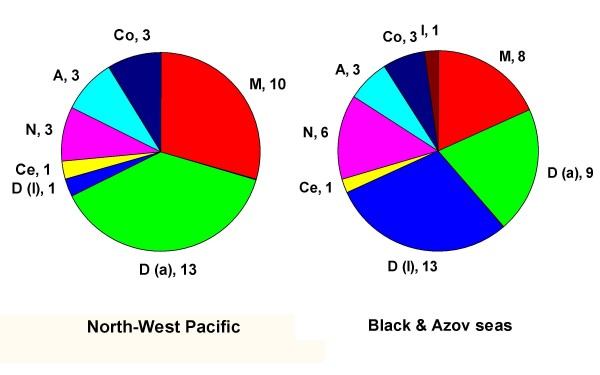
**Higher-level taxonomic structure of the macroparasite fauna of *L. haematocheila *in the Western North Pacific and the Black and Azov seas**. Number of species per higher taxon indicated. *Abbreviations*: A, Acanthocephala; Ce, Cestoda; Co, Copepoda; D (a), Digenea (adult); D (l), Digenea (larval); I, Isopoda; M, Monogenea; N, Nematoda.

The higher-level taxonomic structure of the parasite fauna of *L. haematocheila *in the two distributional ranges is graphically represented in Figure 1. The figure shows the relative representation in terms of number of species of the major parasite taxonomic groupings, namely, Monogenea, Digenea (adult and larval forms separately), Cestoda, Nematoda, Acanthocephala, Copepoda and Isopoda. The species representation is generally similar except for monogeneans which represent a higher proportion of all species in the natural range and digeneans which predominate in the introduced/invasive range of the host. Digeneans also exhibit a reverse pattern with respect to the ratio adult/larval forms, the latter comprising a substantial part of the species list in the introduced/invasive range.

The data in Additional file 1 indicate that *L. haematocheila *has 'lost' 23 species of parasite (3 monogeneans, 13 digeneans, 1 cestode, 3 nematodes, 1 acanthocephalan, and 2 copepods) and 'gained' additional 34 species (1 monogenean, 22 digeneans, 1 cestode, 6 nematodes, 1 acanthocephalan, 2 copepods, and 1 isopod) following its introduction into the Sea of Azov and subsequent invasion of the Black Sea. Whereas problems with the identification may explain the small differences in the monogenean species lists (see below) there appears an apparent trend for replacement of the digenean fauna of *L. haematocheila *after its introduction in the Sea of Azov/Black Sea. Thus, the waretrematine haploporids *Platydidymus flecterotestis *(Zhukov, 1971), *Pseudohapladena mugili *(Zhukov, 1971) and *Skrjabinolecithum spasskii *Belous, 1954 have been replaced in the new distributional range by the haploporine haploporids (*Dicrogaster contracta *Looss, 1902, *Haploporus lateralis *Looss, 1902, *Haploporus *sp., *Saccocoelium obesum *Looss, 1902 and *Saccocoelium tensum *Looss, 1902) and the haplosplanchnids *Haplosplanchnus bivitellosus *Zhukov, 1971, *Hymenocotta mugilis *Wang & Wang, 1993 and *Prohaplosplanchnus diorchis *Tang & Lin, 1978 by *Haplosplanchnus pachysomus *(Eysenhardt, 1829); the latter species groups being characteristic for mullets in the new areas. Furthermore, a large suite of larval forms (13 digenean and 4 nematode species) have been recorded mostly in the Sea of Azov. All these [with the exception of *Hysterothylacium aduncum *(Rudolphi, 1802) and *Acanthostomum imbutiformis *(Molin, 1859)] parasitise fish-eating birds (and some occasionally mammals) as adults. Unfortunately, the above picture is associated with a large number of non-documented records in the introduced/invasive range (*i.e. *lack of evidence for the identification of most adult and all larval digeneans, nematodes, acanthocephalans, copepods and isopods, see Additional file 1).

## Discussion

The taxonomy of the genus *Ligophorus *has not been clarified in substantial detail due to the high morphological similarity of the species; a recently initiated dispute also concerns both species validity and distribution of the monogenean parasites of *L. haematocheila*. Sarabeev & Balbuena [35] described *Ligophorus pilengas *Sarabeev & Balbuena, 2004 from *L. haematocheila *in the Sea of Azov and Black Sea and provided a long list of synonyms of the new species: *L. vanbenedeni *(Parona & Perugia, 1890) of Gusev [36]; *L. chabaudi *Euzet & Suriano, 1977 of Dmitrieva [14], Maltsev & Zhdamirov [19], Maltsev & Miroshnichenko [23], Domnich & Sarabeev [27,30,31], Sarabeev [33], and Sarabeev & Domnich [34]. However, the material reported by Gusev [36] as *L. vanbenedeni *in *L. haematocheila *from Liao-Ho River (Yellow Sea basin) was considered a synonym of *L. chabaudi *by Euzet & Suriano [37]. Miroshnichenko & Maltsev [26] described *L. gusevi *Miroshnichenko & Maltsev, 2004 from *L. haematocheila *in the Black Sea. Balbuena *et al*. [38] did not accept the distinct status of *L. gusevi *which they considered a junior synonym of *L. pilengas *Sarabeev & Balbuena, 2004. Rubtsova *et al. *[39] considered the material reported as *L. chabaudi *by Dmitrieva [14], Sarabeev & Balbuena [35] and Miroshnichenko & Maltsev [26] synonymous with *L. cephali *Rubtsova et al., 2005 and concluded that *L. chabaudi *has not been found in the Black or Azov seas and that the record of this species in the East China Sea of Wu *et al*. [40] needs confirmation due to its zoogeographical incongruence. However, *L. chabaudi *of Dmitrieva [14] has been previously placed in synonymy with *L. pilengas *by Sarabeev & Balbuena [35]; this controversy could be attributed to a nomenclatural error in the lists of synonyms in [35,39]. Sarabeev *et al. *[41] considered that *Ligophorus mugilinus *(Hargis, 1955) is restricted to the Western Atlantic and the Gulf of Mexico. Finally, Dmitrieva *et al. *[42] described a new species from *L. haematocheila*, *L. llewellyni *Dmitrieva, Gerasev & Pron'kina, 2007 and assumed that the material described as *L. pilengas *may actually contain both species; these authors attributed the failure of Sarabeev & Balbuena [35] to discriminate the two species to the insufficient number of measurements used by these authors. Clearly further effort is needed towards clarification of the taxonomic status and distribution of *Ligophorus *spp. in *L. haematocheila*.

The taxonomy of the haploporid digeneans in Additional file 1 is updated following the conclusions of the recent revision by Overstreet & Curran [43]. Two of the three waretrematine haploporids originally described and subsequently reported only in mullets from the Western North Pacific [44,45] were recombined by Overstreet & Curran [43]. These authors found that *Hapalotrema *Zhukov, 1971 is preoccupied by *Hapalotrema *Looss, 1899 (a spirorchiid) and proposed *Platydidymus *Overstreet & Curran, 2005 as the replacement name; *Hapalotrema flecterotestis *Zhukov, 1971 was thus recombined as *Platydidymus flecterotestis *(Zhukov, 1971) Overstreet & Curran, 2005. *Parasaccocoelium mugili *Zhukov, 1971, the type- and only species of the genus *Parasaccocoelium *Zhukov, 1971, was placed by Overstreet & Curran [43] in *Pseudohapladena *Yamaguti, 1952 as *Pseudohapladena mugili *(Zhukov, 1971) Overstreet & Curran, 2005 thus making *Parasaccocoelium *a junior subjective synonym of *Pseudohapladena*. *Dicrogaster contracta *Looss, 1902 was considered a junior synonym of *Dicrogaster perpusilla *Looss, 1902 by Sarabeev & Balbuena [46], a suggestion rejected by Blasco-Costa et al. [47] who redescribed the two species from newly collected Mediterranean material. The identification of *D. contracta *in the Black Sea/Azov Sea region is therefore questionable (Additional file 1).

The list of the records of the bunocotyline hemiurids in Additional file 1 follows the authors' original identification. However, the identification of all materials reported in *L. haematocheila *as *Saturnius papernai *Overstreet, 1977 requires confirmation. Domnich & Sarabeev [28] described *Bunocotyle constrictus *Domnich & Sarabeev, 1999 from *Mugil soiuy *in the Azov Sea, which they later (see *e.g. *[29,32]) considered a misidentification of *S. papernai*. However, Blasco-Costa *et al*. [47] re-examined the type- and voucher material of *B. constrictus *and revealed that all specimens were in poor condition, the egg-size measurements in the original description were erroneous etc. (see [47] for details). Due to these discrepancies, *B. constrictus *and its synonym (*i.e. Saturnius papernai *of Domnich & Sarabeev *inter alia*) was considered a *species inquirenda*. Another doubtful record is of the bunocotyline hemiurid, *Bunocotyle cingulata *Odhner, 1928 in *L. haematocheila *in the Sea of Azov since it appears that a non-documented occurrence of a single worm in mullet fingerlings has been reiterated in a number of papers/abstracts [27-30,32,34].

The systematics of the species of *Diplostomum *Nordmann, 1832 has long been controversial due to the phenotypic plasticity of the adult stage and the simple morphology of the larval stages. Identification of the metacercariae of *Diplostomum *spp. in particular, is practically impossible without experimental completion of the life-cycle due to the paucity of morphological features useful for larval identification and the specific requirements for specimen preparation and examination (see *e.g. *Shigin [48]; Niewiadomska & Niewiadomska-Bugaj [49]). Furthermore, experimental studies have shown that the morphology of the metacercariae of *D*. *paracaudum *(Iles, 1959), *D. pseudospathaceum *Niewiadomska, 1984 and *D. spathaceum *(Rudolphi, 1819) can be affected by the host species, the density of infection, the size of the fish host, and the age of the metacercariae [50-53]. Any of these factors may generate differences between individuals of the same species in natural infections [49]. Therefore, although I have retained authors' identification of *Diplostomum *spp. metacercariae, the record of four species (*i.e*. *D. spathaceum*, *D. paracaudum*, *D. rutili *Razmashkin, 1969 and *D*. *pseudospathaceum *[reported as its synonym *D. chromatophorum *(Brown, 1931)] may depart from the real situation in the region and should be treated with caution (unfortunately all records are non-documented).

A comparison of the parasite faunas of *L. haematocheila *in its native and introduced/invasive range based on the records in the present checklist [14-45,47,54-77] demonstrates that a large number of parasite species was 'lost' in the new distributional range of the host whereas an even greater number was 'gained'. This results in the different higher-level taxonomic structure of the parasite faunas of this host in the two distributional areas and the small number of species (13% of the total list, mostly monogeneans) in common between them. The stepwise introduction of *L. haematocheila *(1978 through 1984) attempted in the Azov Sea basin involved both fry and fish of different ages caught in the Amur and Ussuriisk bays of the Sea of Japan [78]. Therefore, the loss of parasites cannot be entirely attributed to introduced populations derived from uninfected life-history stages; this is supported by the records of most monogenean species (*i.e*. *Gyrodactylus mugili*, *G. zhukovi, Ligophorus kaohsiangsieni*, *L. llewellyni *and *L*. *pilengas*) transferred to the new distributional areas of the host [14,22,35,42].

The replacement of the adult forms (notably the haploporid and haplosplanchnid digeneans) by 'alternative' species of the same families (and, therefore, utilising similar two-host life-cycle strategies with encystment of the cercariae on vegetation) can be considered a change related to the feeding habits of the host. This process appears to reflect biogeographical differences in the 'supply' since opportunities for transmission of the digenean species infecting *L. haematocheila *in the Western North Pacific probably do not exist in the new areas (*e.g. *the absence of the mollusc intermediate host). Further support to the 'supply-side' ecology of parasite transmission provides the 'gain' of the large suite of larval parasite stages (16 species utilising fish as second intermediate and fish-eating birds as definitive hosts). Although this may reflect both high bird and mullet abundance in the areas studied (Sivash, Molochnyi Liman and Obytochna Bay), all Ramsar wetlands of international importance supporting hundred thousands migrating and overwintering birds, I consider these records questionable since none is documented (see also the comment on larval *Diplostomum *spp. identification above).

## Conclusion

Although the present checklist provides information that will facilitate future studies, the interesting question of macroparasite faunal diversity in *L. haematocheila *in its natural and introduced/invasive ranges cannot be dealt with the current data because of unreliability associated with the large number of non-documented and questionable records. This stresses the importance of data quality analysis in using host-parasite database and checklist data.

## Competing interests

The author declares that they have no competing interests.

## Supplementary Material

Additional file 1 Checklist of macroparasites of *Liza haematocheila*. Checklist of helminth and crustacean parasites of *Liza haematocheila *with documented (*) and questionable (?) records marked. *Abbreviations*: MS, *Mugil soiuy*; LH, *L. haematocheila*. Click here for file
