# Supplementary material for: A checklist of macroparasites of Liza haematocheila (Temminck & Schlegel) (Teleostei: Mugilidae)
Source: Parasit Vectors. 2008 Dec 31;1:48. doi: 10.1186/1756-3305-1-48 (PMC2631517; doi:10.1186/1756-3305-1-48)
Supplement: Additional file 1 — Checklist of macroparasites of Liza haematocheila. Checklist of helminth and crustacean parasites of Liza haematocheila with documented (*) and questionable (?) records marked. Abbreviations: MS, Mugil soiuy; LH, L. haematocheila. [file 1756-3305-1-48-S1.doc]

**Additional file 1.** Checklist of helminth and crustacean parasites of *Liza haematocheila* with documented (*) and questionable (?) records marked. *Abbreviations*: MS, *Mugil soiuy*; LH, *L. haematocheila.*

| **Species** | **Host name**  **used** | **Area/Locality** | **Source** |
| --- | --- | --- | --- |
| **MONOGENEA** |  |  |  |
| **Family Dactylogyridae Bychowsky, 1933** |  |  |  |
| *Ligophorus chabaudi* Euzet & Suriano, 1977 |  |  |  |
| *** | MS | China | [40] |
| * | MS | Russian Far East | [14] |
| * | MS | Black Sea | [14] |
|  | MS | Black Sea | [15] |
|  | MS | Black Sea | [19] |
|  | MS | Azov Sea | [20] |
|  | MS | Azov Sea | [21] |
|  | MS | Black Sea | [17] |
|  | MS | Russian Far East | [22] |
|  | MS | Azov Sea | [23] |
|  | MS | Black Sea | [22] |
|  | MS | Azov Sea | [25] |
| * | MS | China | [55] |
|  | MS | Russian Far East | [18] |
|  | MS | Black Sea/Azov Sea | [18] |
|  | MS | Black Sea | [56] |
|  | LH | Black Sea (Crimean coasts) | [57] |
|  | MS | East China Sea | [58] |
| *Ligophorus kaohsianghsieni* (Gusev, 1962) |  |  |  |
| *** | MS | Russian Far East | [36] |
|  | MS | China | [59] |
| * | MS | China | [40] |
| * | MS | Russian Far East | [14] |
| * | MS | Black Sea | [14] |
|  | MS | Black Sea | [16] |
|  | MS | Black Sea | [15] |
|  | MS | Azov Sea | [20] |
|  | MS | Azov Sea | [21] |
|  | MS | Black Sea | [17] |
|  | MS | Russian Far East | [22] |
|  | MS | Azov Sea | [23] |
|  | MS | Black Sea | [22] |
|  | MS | Azov Sea | [27] |
|  | MS | Azov Sea | [29] |
|  | MS | Azov Sea | [30] |
|  | MS | Azov Sea | [25] |
|  | MS | Azov Sea | [33] |
|  | MS | Azov Sea | [34] |
| * | MS | China | [55] |
|  | MS | Russian Far East | [18] |
|  | MS | Black Sea/Azov Sea | [18] |
|  | MS | Black Sea | [56] |
|  | LH | Black Sea | [57] |
|  | MS | East China Sea/South China Sea | [58] |
|  | MS | South China Sea | [58] |
|  | MS | Black Sea | [35] |
|  | MS | Azov Sea | [35] |
|  | LH | Russian Far East | [42] |
| *Ligophorus leporinus* (Zhang & Ji, 1981) |  |  |  |
|  | LH | Russian Far East | [42] |
| *Ligophorus llewellyni* Dmitrieva, Gerasev & Pron’kina, 2007 |  |  |  |
| *** | LH | Russian Far East | [42] |
| *** | LH | Black Sea | [42] |
| *Ligophorus mugilinus* (Hargis, 1955) |  |  |  |
| *** | MS | China | [40] |
| * | MS | China | [55] |
|  | MS | East China Sea | [58] |
| *Ligophorus pilengas* Sarabeev & Balbuena, 2004 |  |  |  |
| (as *L. chabaudi*) | MS | Azov Sea | [27] |
| (as *L. chabaudi*) | MS | Azov Sea | [29] |
| (as *L. chabaudi*) | MS | Azov Sea | [30] |
| (as *L. chabaudi*) | MS | Azov Sea | [31] |
| (as *L. chabaudi*) | MS | Azov Sea | [33] |
| (as *L. chabaudi*) | MS | Azov Sea | [34] |
| *** | MS | Black Sea | [35] |
| *** | MS | Azov Sea | [35] |
| * (as *Ligophorus gussevi* Miroshnichenko & Maltsev, 2004) | MS | Black Sea | [26] |
| * (as *Ligophorus gussevi* Miroshnichenko & Maltsev, 2004) | MS | Azov Sea | [26] |
| *** | LH | Russian Far East | [42] |
| * | LH | Black Sea | [42] |
| *Ligophorus vanbenedeni* (Parona & Perugia, 1890) |  |  |  |
| * | MS | Yellow Sea basin (River Liao Ho) | [36] |
|  | LH | Western Pacific | [60] |
| * | MS | China | [40] |
| ***** | MS | China | [55] |
| **Family Gyrodactylidae van Beneden & Hesse, 1863** |  |  |  |
| *Gyrodactylus mugili* Zhukov, 1970 |  |  |  |
| *** | MS | Sea of Japan (Pos’yet Bay) | [61] |
|  | MS | Sea of Japan | [62] |
|  | MS | Russian Far East | [14] |
|  | MS | Azov Sea | [20] |
|  | MS | Russian Far East | [22] |
|  | MS | Azov Sea | [23] |
|  | MS | Black Sea | [22] |
|  | MS | Azov Sea | [22] |
|  | MS | Azov Sea | [24] |
|  | MS | Azov Sea | [27] |
|  | MS | Azov Sea | [29] |
|  | MS | Azov Sea | [30] |
|  | MS | Azov Sea | [25] |
|  | MS | Azov Sea | [33] |
|  | MS | Azov Sea | [34] |
|  | MS | Russian Far East | [18] |
|  | MS | Black Sea/Azov Sea | [18] |
| *Gyrodactylus zhukovi* Ling, 1962 |  |  |  |
| *** | MS | China | [63] |
|  | MS | Sea of Japan | [62] |
|  | MS | Azov Sea | [20] |
|  | MS | Azov Sea | [21] |
|  | MS | Black Sea | [22] |
|  | MS | Azov Sea | [22] |
|  | MS | Russian Far East | [22] |
|  | MS | Azov Sea | [23] |
| * | MS | Azov Sea | [24] |
|  | MS | Azov Sea | [27] |
|  | MS | Azov Sea | [29] |
|  | MS | Azov Sea | [30] |
|  | MS | Azov Sea | [33] |
|  | MS | Azov Sea | [34] |
|  | MS | Azov Sea | [25] |
| * | MS | China | [55] |
|  | MS | Russian Far East | [18] |
|  | MS | Black Sea/Azov Sea | [18] |
| *Gyrodactylus* sp. (=*Gyrodactylus anguillae* Ergens, 1960?) |  |  |  |
|  | MS | Azov Sea | [23] |
| * | MS | Azov Sea | [24] |
|  | MS | Azov Sea | [25] |
|  | MS | Black Sea/Azov Sea | [18] |
| **Family Microcotylidae Taschenberg, 1879** |  |  |  |
| *Microcotyle mugilis* Vogt, 1878 |  |  |  |
| *** | MS | Russian Far East | [36] |
|  | MS | China | [59] |
|  | MS | Azov Sea | [64] |
|  | MS | Black Sea | [14] |
|  | MS | Black Sea | [15] |
|  | MS | Azov Sea | [21] |
|  | MS | Black Sea | [17] |
|  | MS | Russian Far East | [22] |
|  | MS | Azov Sea | [23] |
|  | MS | Black Sea | [22] |
|  | MS | Azov Sea | [27] |
|  | MS | Azov Sea | [29] |
|  | MS | Azov Sea | [30] |
|  | MS | Azov Sea | [25] |
|  | MS | Azov Sea | [34] |
|  | MS | Russian Far East | [18] |
|  | MS | Black Sea/Azov Sea | [18] |
|  | MS | Black Sea | [56] |
|  | LH | Black Sea | [57] |
| **DIGENEA (adults)** |  |  |  |
| **Family Faustulidae Poche, 1926** |  |  |  |
| *Bacciger lizae* Shen in Shen & Qiu, 1995 |  |  |  |
| *** | LH | Bohai Gulf (China)  East China Sea/Yellow Sea | [65] |
|  | LH | East China Sea/Yellow Sea | [66] |
| *Bacciger mugilis* Shen, 1987 |  |  |  |
| *** | LH | East China Sea/Yellow Sea | [65] |
| **Family Haploporidae Nicoll, 1914** |  |  |  |
| *Dicrogaster contracta* Looss, 1902 |  |  |  |
| ? | MS | Azov Sea | [27] |
| ? | MS | Azov Sea | [32] |
| ? | MS | Azov Sea | [29] |
| ? | MS | Azov Sea | [30] |
| ? | MS | Azov Sea | [33] |
| ? | MS | Azov Sea | [34] |
| ? | MS | Black Sea/Azov Sea | [18] |
| *Haploporus lateralis* Looss, 1902 |  |  |  |
|  | MS | Azov Sea | [27] |
|  | MS | Azov Sea | [32] |
|  | MS | Azov Sea | [29] |
|  | MS | Azov Sea | [30] |
|  | MS | Azov Sea | [25] |
|  | MS | Azov Sea | [33] |
|  | MS | Azov Sea | [34] |
|  | MS | Black Sea/Azov Sea | [18] |
|  | MS | Black Sea | [56] |
| *Haploporus* sp. | MS | Azov Sea | [64] |
| *Platydidymus* *flecterotestis* (Zhukov, 1971) |  |  |  |
| *** | MS | Yellow Sea (River Liao Ho) | [45] |
|  | LH | Bohai Gulf (China) | [67] |
|  | LH | China | [68] |
| * | LH | Bohai Gulf (China)  East China Sea/Yellow Sea | [65] |
|  | MS | Russian Far East | [22] |
|  | MS | Russian Far East | [18] |
|  | LH | East China Sea/Yellow Sea | [66] |
| *Pseudohapladena mugili* (Zhukov, 1971) |  |  |  |
| *** | MS | Sea of Japan (Pos’yet Bay) | [45] |
| * |  | Yellow Sea (River Liao Ho) | [45] |
|  | MS | Russian Far East | [22] |
|  | MS | Russian Far East | [18] |
| *Saccocoelium obesum* Looss, 1902 |  |  |  |
|  | MS | Azov Sea | [27] |
|  | MS | Azov Sea | [32] |
|  | MS | Azov Sea | [34] |
|  | MS | Azov Sea | [29] |
|  | MS | Azov Sea | [30] |
|  | MS | Azov Sea | [33] |
|  | MS | Black Sea/Azov Sea | [18] |
| *Saccocoelium tensum* Looss, 1902 |  |  |  |
|  | MS | Azov Sea | [64] |
|  | MS | Azov Sea | [27] |
|  | MS | Azov Sea | [32] |
|  | MS | Azov Sea | [29] |
|  | MS | Azov Sea | [30] |
|  | MS | Azov Sea | [31] |
|  | MS | Azov Sea | [25] |
|  | MS | Azov Sea | [33] |
|  | MS | Azov Sea | [34] |
|  | MS | Black Sea/Azov Sea | [18] |
|  | MS | Black Sea (off Sevastopol) | [69] |
|  | MS | Black Sea | [56] |
| *Skrjabinolecithum spasskii* Belous, 1954 |  |  |  |
| *** | MS | Primorsk Region (River Suyfun) | [44] |
|  | MS | Russian Far East | [22] |
|  | MS | Russian Far East | [18] |
| Haploporidae gen. sp. | MS | Azov Sea | [25] |
| **Haplosplanchnidae Poche, 1926** |  |  |  |
| *Haplosplanchnus bivitellosus* Zhukov, 1971 |  |  |  |
| *** | MS | Sea of Japan (Pos’yet Bay) | [45] |
| * | MS | Yellow Sea Basin (River Liao Ho) | [45] |
|  | MS | Russian Far East | [22] |
|  | MS | Russian Far East | [18] |
| *Haplosplanchnus pachysomus* (Eysenhardt, 1829) |  |  |  |
|  | MS | Azov Sea | [27] |
|  | MS | Azov Sea | [32] |
|  | MS | Azov Sea | [29] |
|  | MS | Black Sea/Azov Sea | [18] |
|  | MS | Azov Sea | [30] |
|  | MS | Azov Sea | [33] |
|  | MS | Azov Sea | [34] |
| *Hymenocotta mugilis* Wang & Wang, 1993 |  |  |  |
| *** | MS | East China Sea/Yellow Sea | [70] |
|  | MS | Russian Far East | [22] |
| *Prohaplosplanchnus diorchis* Tang & Lin, 1978 |  |  |  |
| * | LH | River Min, Fujian (China) | [71] |
|  | LH | South China Sea | [60] |
| **Family Hemiuridae Looss, 1899** |  |  |  |
| *Bunocotyle cingulata* Odhner, 1928 |  |  |  |
| ? | MS | Azov Sea | [27] |
| ? | MS | Azov Sea | [28] |
| ? | MS | Azov Sea | [32] |
| ? | MS | Azov Sea | [29] |
| ? | MS | Azov Sea | [30] |
| ? | MS | Azov Sea | [34] |
| ? | MS | Black Sea/Azov Sea | [18] |
| *Saturnius overstreeti* Blasco-Costa, Montero, Gibson, Balbuena, Raga, Shvetsova & Kostadinova, 2008 |  |  |  |
| *** | MS | Sea of Japan | [47] |
| *Saturnius papernai* Overstreet, 1977 |  |  |  |
| ?*** (as *Bunocotyle constrictus* Domnich & Sarabeev, 1999 sp. inq.) | MS | Azov Sea (Molochniy Liman) | [28] |
| ? (as *Bunocotyle constrictus* Domnich & Sarabeev, 1999 sp. inq.) | MS | Azov Sea | [27] |
| ? | MS | Azov Sea | [32] |
| ? | MS | Azov Sea | [34] |
| ? | MS | Azov Sea | [29] |
| ? | MS | Azov Sea | [30] |
| ? | MS | Azov Sea | [31] |
| ? | MS | Azov Sea | [33] |
| ? | MS | Black Sea/Azov Sea | [18] |
| ? | MS | Black Sea | [72] |
| ? | LH | Black Sea | [57] |
| *Monolecithotrema lizae* Shen, 1990 |  |  |  |
| *** | LH | East China Sea/Yellow Sea | [73] |
| * | LH | Bohai Gulf (China)  East China Sea/Yellow Sea | [65] |
|  | LH | East China Sea/Yellow Sea | [66] |
| **Family Lecithasteridae Odhner, 1905** |  |  |  |
| *Aponurus lizae* Shen in Shen & Qiu, 1995 |  |  |  |
| *** | LH | Bohai Gulf (China)  East China Sea/Yellow Sea | [65] |
|  | LH | East China Sea/Yellow Sea | [66] |
| *Lecithaster galeatus* Looss, 1907 |  |  |  |
|  | MS | Azov Sea | [27] |
|  | MS | Azov Sea | [32] |
|  | MS | Azov Sea | [29] |
|  | MS | Azov Sea | [30] |
|  | MS | Azov Sea | [33] |
|  | MS | Azov Sea | [34] |
|  | MS | Black Sea/Azov Sea | [18] |
| Lecithasteridae gen. sp. | MS | Azov Sea | [25] |
| **Opecoelidae Stunkard, 1931** |  |  |  |
| *Podocotyle lizae* Qiu & Liang in Shen & Qiu, 1995 |  |  |  |
| *** | LH | Bohai Gulf (China)  East China Sea/Yellow Sea | [65] |
|  | LH | East China Sea/Yellow Sea | [66] |
| *Podocotyle reflexa* (Creplin, 1825) | LH | Sea of Japan | [62] |
| Halipegidae gen. sp. | MS | Azov Sea | [25] |
| **DIGENEA (larval)** |  |  |  |
| **Acanthostomidae** Poche, 1926 |  |  |  |
| *Acanthostomum imbutiformis* (Molin, 1859) |  |  |  |
| ? | MS | Azov Sea | [27] |
| ? | MS | Azov Sea | [32] |
| ? | MS | Azov Sea | [29] |
| ? | MS | Azov Sea | [30] |
| ? | MS | Azov Sea | [31] |
| ? | MS | Azov Sea | [33] |
| ? | MS | Azov Sea | [34] |
| **Diplostomidae Poirier, 1886** |  |  |  |
| ? *Diplostomum paracaudum* (Iles, 1959) | MS | Azov Sea | [25] |
| *Diplostomum pseudospathaceum* Niewiadomska, 1984 [as *D. chromatophorum* (Brown, 1931)] |  |  |  |
| ? | MS | Azov Sea | [27] |
| ? | MS | Azov Sea | [32] |
| ? | MS | Azov Sea | [29] |
| ? | MS | Azov Sea | [30] |
| ? | MS | Azov Sea | [31] |
| ? | MS | Azov Sea | [33] |
| ? | MS | Azov Sea | [34] |
| ? | MS | Black Sea/Azov Sea | [18] |
| *Diplostomum rutili* Razmashkin, 1969 |  |  |  |
| ? | MS | Azov Sea | [27] |
| ? | MS | Azov Sea | [32] |
| ? | MS | Azov Sea | [29] |
| ? | MS | Azov Sea | [30] |
| ? | MS | Azov Sea | [34] |
| ? | MS | Black Sea/Azov Sea | [18] |
| *Diplostomum spathaceum* (Rudolphi, 1819) |  |  |  |
| ? | MS | Azov Sea | [27] |
| ? | MS | Azov Sea | [32] |
| ? | MS | Azov Sea | [29] |
| ? | MS | Azov Sea | [30] |
| ? | MS | Azov Sea | [34] |
| ? | MS | Black Sea/Azov Sea | [18] |
| *Diplostomum* sp. |  |  |  |
|  | MS | Azov Sea | [64] |
|  | MS | Azov Sea | [27] |
|  | MS | Azov Sea | [32] |
|  | MS | Azov Sea | [29] |
|  | MS | Azov Sea | [25] |
|  | MS | Black Sea/Azov Sea | [18] |
|  | MS | Black Sea | [56] |
| *Posthodiplostomum brevicaudatum* (Nordmann, 1832) |  |  |  |
| ? | MS | Azov Sea | [27] |
| ? | MS | Azov Sea | [32] |
| ? | MS | Azov Sea | [29] |
| ? | MS | Azov Sea | [30] |
| ? | MS | Azov Sea | [34] |
| ? | MS | Black Sea/Azov Sea | [18] |
| *Tylodelphys clavata* (Nordmann, 1832) |  |  |  |
| ? | MS | Azov Sea | [27] |
| ? | MS | Azov Sea | [32] |
| ? | MS | Azov Sea | [29] |
| ? | MS | Azov Sea | [30] |
| ? | MS | Azov Sea | [25] |
| ? | MS | Azov Sea | [34] |
| ? | MS | Black Sea/Azov Sea | [18] |
| ? | MS | Black Sea | [56] |
| **Echinostomatidae Looss, 1899** |  |  |  |
| *Stephanoprora* sp. (as *Mesorchis* sp.) |  |  |  |
| ? | MS | Azov Sea | [27] |
| ? | MS | Azov Sea | [32] |
| ? | MS | Azov Sea | [29] |
| ? | MS | Azov Sea | [34] |
| ? | MS | Black Sea/Azov Sea | [18] |
| **Heterophyidae Odhner, 1914** |  |  |  |
| *? Ascocotyle coleostoma* (Looss, 1896) | MS | Azov Sea | [29] |
| *Ascocotyle* (*Phagicola*) *sinoecum* Ciurea, 1933 |  |  |  |
| ? | MS | Azov Sea | [27] |
| ? | MS | Azov Sea | [32] |
| ? | MS | Azov Sea | [29] |
| ? | MS | Azov Sea | [30] |
| ? | MS | Azov Sea | [31] |
| ? | MS | Azov Sea | [33] |
| ? | MS | Azov Sea | [34] |
| ? | MS | Black Sea/Azov Sea | [18] |
| *Cryptocotyle concavum* (Creplin, 1825) |  |  |  |
| ? | MS | Azov Sea | [27] |
| ? | MS | Azov Sea | [32] |
| ? | MS | Azov Sea | [30] |
| ? | MS | Azov Sea | [34] |
| ? | MS | Black Sea/Azov Sea | [18] |
| *Heterophyes nocens* Onji & Nishio, 1916 |  |  |  |
|  | LH | Japan | [62] |
|  | LH | Japan | [74] |
| *Pygidiopsis genata* Looss, 1907 |  |  |  |
| ? | MS | Azov Sea | [27] |
| ? | MS | Azov Sea | [32] |
| ? | MS | Azov Sea | [29] |
| ? | MS | Azov Sea | [30] |
| ? | MS | Azov Sea | [34] |
| ? | MS | Black Sea/Azov Sea | [18] |
| Heterophyidae gen. sp. |  |  |  |
|  | MS | Azov Sea | [27] |
|  | MS | Azov Sea | [32] |
|  | MS | Azov Sea | [29] |
|  | MS | Azov Sea | [34] |
|  | MS | Black Sea/Azov Sea | [18] |
| **CESTODA (larval)** |  |  |  |
| ** Diphyllobothrium latum* (Linnaeus, 1758) | LH | South Korea | [75] |
| ** Ligula* sp. | MS | Black Sea/Azov Sea | [76] |
| **NEMATODA** |  |  |  |
| **Family Acuariidae Railliet, Henry & Sisoff, 1912** |  |  |  |
| *Cosmocephalus obvelatus* (Creplin, 1825) (larva) |  |  |  |
| ? | MS | Azov Sea | [29] |
| ? (as *Cosmocephalus obvelatus magnus* Wassilkowa, 1926) | MS | Azov Sea | [30] |
| ? | MS | Black Sea/Azov Sea | [18] |
| **Family Anisakidae (Railliet & Henry, 1912)** |  |  |  |
| *Contracaecum microcephalum* (Rudolphi, 1819) (larva) |  |  |  |
| ? | MS | Azov Sea | [29] |
| ? | MS | Azov Sea | [30] |
| ? | MS | Azov Sea | [33] |
| ? | MS | Black Sea/Azov Sea | [18] |
| *Contracaecum* sp. (larva) |  |  |  |
| ? | MS | Azov Sea | [27] |
| ? | MS | Azov Sea | [25] |
| ? | MS | Azov Sea | [34] |
| *Hysterothylacium aduncum* (Rudolphi, 1802) | LH | Black Sea | [57] |
| **Family Capillariidae Railliet, 1915** |  |  |  |
| *Pseudocapillaria tomentosa* (Dujardin, 1843) (as *Capillaria tomentosa*) | MS | Black Sea | [56] |
| **Family Cucullanidae Cobbold, 1864** |  |  |  |
| *Cuculanus mugili* Belous, 1965 |  |  |  |
| *** | MS | River Tavrichanka (Primorsk Region) | [77] |
|  | MS | Russian Far East | [22] |
|  | MS | Russian Far East | [18] |
| *Cucullanus spirocaudus* Lee, 1984 | LH | East China Sea/Yellow Sea | [66] |
| *Dichelyne minutus* (Rudolphi, 1819) | LH | Black Sea | [57] |
| **Family Philometridae Baylis & Daubney, 1926** |  |  |  |
| *Philometra biglobocerca* Belous, 1965 |  |  |  |
| *** | MS | River Suyfun (Primorsk Region) | [77] |
|  | MS | Russian Far East | [18] |
| Nematoda gen. sp. | MS | Black Sea | [56] |
| **ACANTHOCEPHALA** |  |  |  |
| **Family Echinorhynchidae Cobbold, 1876** |  |  |  |
| *Acanthocephalus luzus* Li (?) | LH | East China Sea/Yellow Sea | [66] |
| **Family Neoechinorhynchidae Ward, 1917** |  |  |  |
| *Neoechinorhynchus agilis* (Rudolphi, 1819) |  |  |  |
|  | MS | River Liao He (China) | [78] |
|  | MS | Azov Sea | [27] |
|  | MS | Azov Sea | [29] |
|  | MS | Azov Sea | [30] |
|  | MS | Azov Sea | [33] |
|  | MS | Azov Sea | [34] |
|  | MS | Black Sea/Azov Sea | [18] |
|  | MS | Black Sea | [56] |
| *Neoechinorhynchus* sp. | MS | Azov Sea | [25] |
| **Family Quadrigyridae Van Cleave, 1920** |  |  |  |
| *Acanthogyrus* (*Acanthosentis*) *tylosuri* (Yamaguti, 1939) |  |  |  |
| *** | MS | River Liao He (China) | [78] |
| (as *Neoechinorhynchus tylosuri* Yamaguti, 1939) | MS | Russian Far East | [22] |
|  | MS | Black Sea | [22] |
|  | MS | Azov Sea | [22] |
| (as *Neoechinorhynchus tylosuri* Yamaguti, 1939) | MS | Russian Far East | [18] |
| (as *Neoechinorhynchus tylosuri* Yamaguti, 1939) | MS | Black Sea/Azov Sea | [18] |
| **COPEPODA** |  |  |  |
| **Family Caligidae Latreille, 1829** |  |  |  |
| *Caligus orientalis* Gusev, 1951 |  |  |  |
|  | MS | Russian Far East | [22] |
|  | MS | Russian Far East | [18] |
| **Family Ergasilidae Edwards, 180** |  |  |  |
| *Ergasilus nanus* van Beneden, 1871 | MS | Black Sea | [56] |
| *Ergasilus* sp. | MS | Azov Sea | [25] |
| **Family Lernanthropidae Kabata, 1979** |  |  |  |
| *Lernanthropus mugilis* Brian, 1898 | MS | Russian Far East | [22] |
| *Lernanthropsis mugili* (Shishido, 1898) | MS | Pacific Soviet Union | [62] |
|  | MS | Russian Far East | [18] |
| **ISOPODA** |  |  |  |
| **Family Cymothoidae Dana, 1852** |  |  |  |
| *Lironeca taurica* Czerniavsky, 1868 | MS | Azov Sea | [25] |
